# The equity catastrophe: how securitized financing collapses essential care during health crises

**DOI:** 10.1093/haschl/qxag042

**Published:** 2026-02-20

**Authors:** Alpha Umaru Bai-Sesay, Jusu Musa, Daniel Karim Dauda Sesay

**Affiliations:** Public Health Surveillance, Ministry of Health/National Public Health Agency, Freetown, Sierra Leone; Research and Scientific Division, Sustainable Health Systems, Freetown, Sierra Leone; Research and Scientific Division, Insights for Development Impact, Freetown, Sierra Leone; Public Health Surveillance, Ministry of Health/National Public Health Agency, Freetown, Sierra Leone; Research and Scientific Division, Insights for Development Impact, Freetown, Sierra Leone; Public Health Surveillance, Ministry of Health/National Public Health Agency, Freetown, Sierra Leone; Research and Scientific Division, Insights for Development Impact, Freetown, Sierra Leone

**Keywords:** health equity, health security financing, primary health care, decentralization, continuity of care

## Abstract

Global health systems have demonstrated strong capacity for rapid outbreak containment. However, across recent health emergencies, including Ebola, COVID-19, and Mpox. These responses have repeatedly been associated with large-scale disruptions to routine essential health services and excess nonoutbreak mortality, particularly among women and children. This article examines how prevailing pandemic preparedness policies and financing arrangements can be redesigned to achieve effective outbreak containment while maintaining continuity of essential primary health care. Drawing on documented experiences across multiple outbreaks and settings, the analysis traces a consistent causal pathway through which interpretations of International Health Regulations obligations and donor financing requirements prioritize centralized containment capacities without corresponding safeguards for peripheral service delivery. These policy and financing priorities trigger resource reallocation away from community-level services, reduce health care utilization, and contribute to preventable mortality from routine causes. We describe this recurring pattern as an “Equity Catastrophe,” reflecting its disproportionate impact on socioeconomically marginalized populations during health emergencies. The article identifies key structural drivers of this outcome and proposes three targeted policy reforms aimed at strengthening preparedness while protecting essential health services. These recommendations are directed at national governments, global normative institutions, and international financing partners involved in pandemic preparedness and response.

Key Points

- Centralized, containment-focused health security funding drives a predictable “equity catastrophe,” causing high, preventable nonoutbreak mortality, particularly in Reproductive, Maternal, Newborn, Child, and Adolescent Health.
- New global and national health security frameworks must transition from purely technical metrics to measurable equity metrics focused on maintaining essential services for vulnerable populations.
- Policies must shift funding to decentralize preparedness by investing in community health worker programs and redundant local supply chains to ensure continuity of primary care.

## Introduction

The global community has repeatedly demonstrated an impressive, reactive capacity for outbreak containment.^1-3^ Yet, in every major health crisis of the last decade, a recurring systemic failure has been evident: the large-scale collapse of routine essential health services, with predictable excess mortality among women and children.^4-6^ The objective of this article is not to argue against outbreak containment but to examine how current pandemic preparedness policies and financing structures can be redesigned to achieve effective containment while simultaneously protecting continuity of essential primary health care (PHC) services.

Specifically, this failure arises from the way global and national preparedness policies interpret and operationalize International Health Regulations (IHR) obligations and donor financing requirements in a manner that prioritizes centralized containment capacities without corresponding safeguards for peripheral PHC continuity.^7^

In this article, we refer to this recurring pattern as an “Equity Catastrophe”: a policy-driven surge in preventable, nonoutbreak mortality that disproportionately affects socioeconomically marginalized populations during health emergencies. Across outbreaks, excess nonoutbreak mortality follows a consistent causal pathway: preparedness policies and financing trigger centralized resource reallocation, which disrupts peripheral service delivery, leading to declines in care utilization and preventable deaths. During the Ebola response, this pattern was associated with massive spikes in maternal and neonatal deaths due to facility closure. The COVID-19 response led to staggering global drops in childhood immunization, generating subsequent lethal outbreaks of measles and cholera.^8,9^ Even smaller-scale responses, such as for Mpox, have demonstrated how shifts in focus disproportionately disrupt basic sexual and reproductive health services for specific vulnerable groups.^8,10^ These patterns have been documented across diverse settings and outbreak scales, suggesting a systemic rather than context-specific failure. This article argues that the recurrent, predictable loss of Reproductive, Maternal, Newborn, Child, and Adolescent Health (RMNCAH) lives during health emergencies reflects structural harm arising from policy design and financing choices that amplify pre-existing socioeconomic inequities. The analysis is directed at three policy audiences: global normative bodies responsible for preparedness frameworks (including WHO and IHR governance), national governments designing and implementing National Action Plans for Health Security (NAPHS), and major bilateral and multilateral financing institutions that shape preparedness incentives. We identify the mechanisms of this diversion and propose actionable reforms for global normative institutions, national governments, and international donors to better align outbreak response with the preservation of essential health services. The analysis proceeds in three parts: (1) examining the operational pathway through which securitized financing triggers the collapse of peripheral care; (2) critiquing the global financial architecture that incentivizes this outcome; and (3) proposing three targeted policy shifts designed to build health systems capable of effective containment while maintaining continuity of care for vulnerable populations.

## The anatomy of the equity catastrophe

The recurrent failure to protect essential services during a crisis is not an operational lapse but a systemic design flaw rooted in the inherent bias toward centralized rapid response, which consistently increases the risk of large-scale disruption to essential services during health emergencies.^5,10^ When a health emergency is declared, the policy response is an immediate resource diversion driven by prevailing interpretations of IHR core capacity requirements and donor financing conditionalities, wherein financial and human resources are systematically stripped from peripheral PHC facilities and rerouted to high-profile central functions.^5,10,11^ This often results in the redeployment of RMNCAH personnel, including midwives and specialized nurses, from peripheral facilities to outbreak treatment centers and centralized surveillance functions.^12^ The sudden loss of skilled staff at the facility level leads to service suspension or severe reductions in maternal, newborn, and child health care availability. Simultaneously, critical logistics and supplies (such as Personal Protective Equipments (PPE), RMNCAH medicines, and vehicle fuel) are instantly prioritized for centralized containment, interrupting routine supply chains and leading to preventable stockouts of life-saving commodities at peripheral clinics, a pattern seen across crises like Ebola and COVID-19.^12-14^ These response policies then disproportionately affect socioeconomically vulnerable populations, layering barriers onto vulnerable populations; centralized resource deployment making high-quality care physically and financially inaccessible, particularly in rural and low-income settings where transportation constraints and movement restrictions are most severe.^12-14^ Furthermore, top-down, often security-enforced containment measures have been associated with declines in community trust in the formal health system, leading to a long-term trust deficit that subsequently obstructs other public health efforts.^15,16^ The ultimate result is the devastating nonoutbreak mortality surge, the true Equity Catastrophe which confirms that the policy-driven choice to prioritize centralized security over decentralized resilience contributes to repeated failures to protect the lives of women and children from preventable, routine causes.^12,13^ Understanding this operational pathway is essential for evaluating why current preparedness financing frameworks fail to reward continuity of care, and for designing corrective policy reforms. This systemic mechanism, where centralized triggers lead inexorably to peripheral collapse and excess mortality, is summarized in Table 1.

**Table 1.** The anatomy of the equity catastrophe: how securitized response mechanisms drive collateral mortality

Policy trigger (during crisis)	Immediate operational consequence	Impact on peripheral PHC	Aggravated equity barrier	Resultant “equity catastrophe” outcome
**1. Centralized resource diversion:** Mandated by IHR/donor priorities, pulling financial and human capital to national-level response.	Redeployment of skilled RMNCAH staff (midwives, nurses) to outbreak treatment centers/surveillance.	Sudden loss of local clinical capacity; closure or severe reduction of maternal and child health services.	**Physical and financial inaccessibility:** High-quality care moves to distant, centralized locations, impossible for low-income/rural populations to reach, especially under travel restrictions.	**Direct excess mortality:** Preventable maternal deaths, stillbirths, and neonatal deaths due to lack of skilled birth attendance and emergency obstetric care.
**2. Centralized logistics prioritization:** PPE, vehicles, fuel, and supply chains are commandeered for containment efforts.	Interruption of routine medical supply chains (vaccines, oxytocin, and antibiotics).	Stockouts of life-saving commodities at community clinics and health posts.	**Commodity deprivation:** Even when a facility is open, essential medicines and vaccines are unavailable, rendering care ineffective.	**Indirect excess mortality:** Deaths from vaccine-preventable diseases (measles and cholera), postpartum hemorrhage, and childhood infections due to lack of prophylaxis and treatment.
**3. Top-down security enforcement:** Quarantines, curfews, and fear-based messaging to ensure containment.	Erosion of community trust in the formal health system; fear of facilities seen as outbreak sources.	Collapse in health-seeking behavior; avoidance of facilities for routine, preventive, and urgent care.	**Trust deficit:** Community disengagement creates a long-term barrier to all public health programs, extending the crisis impact.	**Systemic degradation:** Undermines decades of PHC investment; mortality surge becomes a durable feature of postcrisis recovery.

## The flawed architecture of securitized financing and the path to proequity resilience

The structural flaw in the response mechanism is mirrored and reinforced by the global health financing architecture, which shapes funding flows in ways that can reinforce the service disruptions described above.^10,17^ A central design feature of the current financing architecture is the prioritization of outbreak containment capacities over investments in routine service continuity.^18^ Since the early 2000s, the securitization of health, viewing threats through a national security lens to protect borders has led to financial instruments designed for centralized containment rather than local service delivery and equity.^10^ The current financial ecosystem, including initiatives like the Global Health Security Agenda and the Pandemic Fund, is heavily weighted toward IHR core capacities, which, while essential, are often operationalized through nationally centralized investments in surveillance, laboratories, and emergency operations centers that inherently conflicts with local resilience.^10,18,19^ These financing mechanisms favor investments that are rapidly deployable, nationally visible, and easily attributable to preparedness benchmarks; while providing limited incentives to fund decentralized primary care systems whose benefits are less immediately observable during emergencies. Current models lack financial incentives for maintaining continuity of care at the community level, instead rewarding rapid, visible deployment of technology and centralized teams over sustained, quiet investment in peripheral PHC structures.^10,18^ This preference for large-scale national contracts bypasses the critical need for sustained investment in Community Health Worker (CHW) systems, local drug buffer stocks, and essential clinic maintenance, ensuring the PHC foundation remains fragile and vulnerable to shock.^20^ Concurrently, The indirect health costs associated with service disruption, including excess maternal, neonatal, and child mortality are largely absent from preparedness financing metrics and accountability frameworks.^10,13^ This omission is a policy failure rooted in measurement, ensuring that RMNCAH deaths are treated as a mere “externality,” guaranteeing that the centralized response system repeats its mistakes. Therefore, misaligned with the goal of service continuity, as it tends to reward centralized response capacities while insufficiently protecting peripheral service delivery during crises.

Because NAPHS serve as the primary vehicle through which countries translate preparedness priorities into financed activities, the design of this financing architecture directly shapes whether NAPHS investments strengthen centralized response alone or also protect peripheral primary care systems. To correct this flawed architecture, policy must urgently shift from funding security over health to funding resilience through health. Our three interlocking policy imperatives to achieve this shift are detailed in Table 2 and elaborated below.

1. Policy imperatives: financing proequity resilience

**Table 2.** From securitized collapse to proequity resilience: a policy reform framework.

Policy imperative	Core reform action	Key mechanism for change	Financing and accountability lever	Expected proequity resilience dividend
1. Mandate decentralized investment	Legislate ring-fenced funding (eg, ≥30%) within National Action Plans for Health Security for local, peripheral system resilience.	**Conditionality:** Major donors (eg, United States, Pandemic Fund) approve NAPHS funding contingent on this allocation.	**Financing shift:** Directs preparedness money away from solely central-level assets toward the PHC frontline.	**Antifragile PHC:** CHWs maintain trust and RMNCAH service continuity during lockdowns. Local buffer stocks insulate clinics from national supply chain failure, preventing stockouts.
		**Targets:** (i) Guaranteed salaries and training for Community Health Workers. (ii) Redundant subnational (Tier 3) buffer stocks for RMNCAH commodities.	**Accountability:** NAPHS approval and disbursement milestones tied to verified local investments.	
2. Embed measurable equity metrics	Adopt the **continuity of care resilience index** and mandate wealth-quintile disaggregation for all essential service data.	**Core indicator:** CCRI = (Utilization in lowest wealth quintile at crisis peak)/(Baseline utilization). Target: CCRI ≈ 1.	**New success metric:** Moves beyond IHR technical compliance to measure human outcome equity.	**Accountability for protection:** Prevents equity impacts from being ignored as externalities. Creates political and financial incentives to protect service continuity for the poor.
		**Mandatory transparency:** All reporting to donors (bilateral, pandemic fund) must disaggregate service utilization by income, gender, and geography.	**Accountability:** Makes the “invisible costs” of RMNCAH mortality visible and attributable to policy choices, forcing course-correction.	
3. Integrate preparedness and PHC	Legislate the merger of “health security” and “PHC strengthening” budget lines and planning processes.	**Redefinition:** Frameworks preparedness as the **inherent shock-absorption capacity of the PHC system**, not a separate technical program.	**Synergistic financing:** Every dollar spent on “security” inherently strengthens the system that serves the most vulnerable daily.	**Compound returns on investment:** Creates a resilient, equitable system that is more effective for both routine care *and* crisis response. Achieves true health security by securing the well-being of the most marginalized.
		**Dual-use investment:** CHW training for outbreak surveillance *also* enhances RMNCAH outreach; local logistics for epidemic response *also* strengthens routine vaccine supply chains.	**Institutional alignment:** Breaks down programmatic silos at ministry and donor levels.	

The policy objective should shift from mere critique to creating an inherently resilient and equitable health security system by correcting the perverse incentives and structural biases embedded in current global financing mechanisms. This requires mandating decentralized investment via NAPHS, which guides national preparedness investments and unlocks international funding. Specifically, the United States and major multinational donors must condition NAPHS funding approval on a clear commitment to ring-fencing a defined and transparent proportion of preparedness funding for local, peripheral infrastructure resilience through existing financing approval and performance review mechanisms. This dedicated funding must target two areas critical for protecting RMNCAH continuity of care: first, it must be used to guarantee CHW salaries, training, and supervision, as CHW systems are the only layer reliably maintaining trust and engagement during security lockdowns and integrating them into both RMNCAH delivery and outbreak surveillance, strengthens the system's ability to maintain service delivery under stress; second, it must mandate the financing of redundant, subnational (Tier 3) depots for essential RMNCAH commodities (like vaccines and oxytocin), shifting the focus from a single, failure-prone central warehouse to multiple resilient local nodes that insulate peripheral clinics from national or global supply chain shocks.

1. Embed measurable equity metrics for accountability

Moving beyond the insufficient technical metrics that fail to capture the human cost of the Equity Catastrophe, policy must evolve to include explicit, measurable equity metrics that ensure accountability for the protection of vulnerable populations. We propose piloting a quantifiable metric, the Continuity of Care Resilience Index (CCRI) as a core policy indicator for all preparedness plans. The CCRI measures the differential change in utilization of key essential services across the lowest-income wealth quintile from a baseline to the emergency peak; a high-performing system is one where the CCRI is close to 1 (meaning utilization did not drop significantly).^21^ This metric is presented as an illustrative example of how continuity of care could be systematically measured, rather than as a prescriptive global standard. To support this, all funding agreements, from the Pandemic Fund to bilateral aid, should require routine data disaggregation for both direct outbreak outcomes and secondary service disruptions by gender, geography (rural/urban), and income quintile. This mandatory data directly links the loss of RMNCAH services to the policy choices made, preventing the “invisible costs” of the catastrophe from being ignored and forcing policymakers to confront the human cost of their centralized response.

1. Integrate preparedness and PHC

A critical policy shift requires abandoning the silo between “health security” and “primary health care strengthening,” viewing them instead as two sides of the same coin. Policy must align preparedness and PHC planning and financing processes for routine PHC strengthening and outbreak preparedness, reframing preparedness not as an add-on, but as the core ability of the PHC system to absorb and manage shocks without collapsing. This integration can generate substantial equity benefits where investments in CHW training for outbreak surveillance simultaneously increase capacity for routine RMNCAH outreach, and investments in reliable local logistics for epidemic response also strengthen the supply chain for routine childhood vaccines. This approach ensures that every dollar spent on security also strengthens the system that serves the most vulnerable every day. Policy success is Proequity Resilience, a preparedness posture where the response to an outbreak is managed locally, sustains essential services, and is intrinsically fair, moving the global agenda beyond simply securing borders to securing the well-being of the most marginalized citizens. Together, these reforms address the financing incentives and accountability gaps identified earlier by linking preparedness success to the ability of health systems to sustain essential services during crises

## Conclusion

Evidence from the past decade indicates that prevailing global health security policies and financing arrangements, which emphasize centralized containment, are associated with repeated disruptions to essential health services during crises. The staggering, preventable excess mortality in RMNCAH during outbreaks like Ebola and COVID-19 is not an unavoidable tragedy; it reflects the downstream effects of policy and financing choices that prioritize outbreak control without sufficient safeguards for service continuity that prioritizes detection over delivery, and containment over continuity. This article has shown that repeated disruptions to primary health services during emergencies arise from identifiable policy design and financing gaps. By favoring centralized, technical capacities and insufficiently accounting for the role of decentralized PHC in absorbing shocks, the financial architecture actively rewards the very process that marginalizes the poor.

To strengthen global health security, policy frameworks should evolve to better align outbreak preparedness with the protection of essential health services based on three urgent imperatives: (1) Financial mandate for decentralization: International funding must be conditioned on the mandatory allocation of a significant percentage of preparedness funds (eg, 30%) within NAPHS to fortify the local, peripheral system, specifically through CHW integration and redundant local supply chain buffers. (2) Accountability through equity metrics: The focus must transition from IHR technical compliance to measurable human outcomes. The adoption of a CCRI and mandatory disaggregation of service utilization data by wealth quintile will force policymakers to confront and account for the equity impacts of their response strategies. (3) Integration of security and PHC: Preparedness must be redefined not as a separate entity but as the inherent shock-absorption capacity of the PHC system. Merging these policy and financial silos ensures that investment in security simultaneously strengthens the routine services that women and children depend on every day.

The global health policy community must recognize that security is inseparable from equity. A health system is only as prepared as its most vulnerable community is served. Moving forward, preparedness efforts that succeed in containing outbreaks while sustaining essential services for vulnerable populations offer a more complete and durable measure of health system readiness, a commitment that secures not only global borders but also the lives of the most marginalized citizens during crisis.

## Supplementary Material

qxag042_Supplementary_Data
